# Prediction of persistent incomplete occlusion of intracranial aneurysms treated with woven EndoBridge device

**DOI:** 10.1007/s10143-025-03439-8

**Published:** 2025-03-22

**Authors:** Muhammed Amir Essibayi, Mohamed Sobhi Jabal, Hasan Jamil, Hamza Adel Salim, Basel Musmar, Nimer Adeeb, Mahmoud Dibas, Nicole M. Cancelliere, Jose Danilo Bengzon, Oktay Algin, Sherief Ghozy, Sovann V. Lay, Adrien Guenego, Leonardo Renieri, Joseph Carnevale, Guillaume Saliou, Panagiotis Mastorakos, Kareem El Naamani, Eimad Shotar, Markus Möhlenbruch, Michael Kral, Charlotte Chung, Mohamed M. Salem, Ivan Lylyk, Paul M. Foreman, Hamza Shaikh, Vedran Župančić, Muhammad U. Hafeez, Joshua Catapano, Muhammad Waqas, Muhammed Said Besler, Yasin Celal Gunes, James D. Rabinov, Julian Maingard, Clemens M. Schirmer, Mariangela Piano, Anna L. Kühn, Caterina Michelozzi, Robert M. Starke, Ameer Hassan, Mark Ogilvie, Anh Nguyen, Jesse Jones, Waleed Brinjikji, Marie T. Nawka, Marios Psychogios, Christian Ulfert, Bryan Pukenas, Jan-Karl Burkhardt, Thien Huynh, Juan Carlos Martinez-Gutierrez, Sunil A. Sheth, Diana Slawski, Rabih Tawk, Benjamin Pulli, Boris Lubicz, Pietro Panni, Ajit S. Puri, Guglielmo Pero, Eytan Raz, Christoph J. Griessenauer, Hamed Asadi, Adnan Siddiqui, Elad I. Levy, Deepak Khatri, Neil Haranhalli, Andrew F. Ducruet, Felipe C. Albuquerque, Robert W. Regenhardt, Christopher J. Stapleton, Peter Kan, Vladimir Kalousek, Pedro Lylyk, Srikanth Boddu, Jared Knopman, Stavropoula I. Tjoumakaris, Hugo H. Cuellar-Saenz, Pascal M. Jabbour, Frédéric Clarençon, Nicola Limbucci, Vitor Mendes Pereira, Aman B. Patel, David J. Altschul, Adam A. Dmytriw

**Affiliations:** 1 Departments of Neurological Surgery, Radiology, and Montefiore-Einstein Cerebrovascular Research Lab, Montefiore Medical Center, Albert Einstein College of Medicine, Bronx, NY USA; 2 Department of Radiology, Division of Neuroradiology, Johns Hopkins Medical Center, Baltimore, MD USA; 3 Department of Neurosurgery and Interventional Neuroradiology, Louisiana State University, Shreveport, LA USA; 4 Neurovascular Centre, Divisions of Therapeutic Neuroradiology and Neurosurgery, St. Michael’s Hospital, University of Toronto, Toronto, ON Canada; 5 Department of Radiology, Medical Faculty, Ankara University, Ankara, Turkey; 6 Departments of Radiology and Neurosurgery, Mayo Clinic, Rochester, MN USA; 7 Department of Neuroradiology, Centre Hospitalier de Toulouse, Toulouse, France; 8 Department of Neuroradiology, Hôpital Universitaire Erasme, Brussels, Belgium; 9 Department of Neuroradiology, Ospedale Careggi Di Firenze, Florence, Italy; 10 Department of Neurosurgery and Neuroradiology, Weill Cornell School of Medicine, New York Presbyterian Hospitaland , New York, NY USA; 11 Department of Neuroradiology, Centre Hospitalier Vaudois de Lausanne, Lausanne, Switzerland; 12 Department of Neurosurgery, Thomas Jefferson University Hospital, Philadelphia, PA USA; 13 Department of Neuroradiology, Hôpital Pitié-Salpêtrière, Paris, France; 14 Department of Neuroradiology, Universitätsklinikum Heidelberg, Heidelberg, Germany; 15 Department of Neurosurgery, Christian Doppler University Hospital & Institute of Neurointervention, Salzburg, Austria; 16 Departments of Radiology & Neurosurgery, NYU Langone Health Center, New York, NY USA; 17 Department of Neurosurgery, University of Pennsylvania Medical Center, Philadelphia, PA USA; 18 Department of Neuroradiology, Clínica La Sagrada Familia, Buenos Aires, Argentina; 19 Department of Neurosurgery, Orlando Health Neuroscience and Rehabilitation Institute, Orlando, FL USA; 20 Department of Neuroradiology, Clinical Hospital Center ‘Sisters of Mercy’, Zagreb, Croatia; 21 Department of Neurosurgery, UTMB and Baylor School of Medicine, Houston, TX USA; 22 Department of Neurosurgery, Barrow Neurological Institute, Phoenix, AZ USA; 23 Department of Neurosurgery, State University of New York at Buffalo, Buffalo, NY USA; 24 Department of Radiology, Kahramanmaraş Necip Fazıl City Hospital, Kahramanmaraş, Türkiye; 25 Department of Radiology, Kırıkkale Yuksek Ihtisas Hospital, Kırıkkale, Türkiye; 26 Neuroendovascular Program, Massachusetts General Hospital, Harvard University, Boston, MA USA; 27 Department of Neuroradiology, Austin Health, Heidelberg, VIC Australia; 28 Department of Neurosurgery and Radiology, Geisinger Hospital, Danville, PA USA; 29 Department of Neuroradiology, Ospedale Niguarda Cà Granda, Milan, Italy; 30 Department of Neuroradiology, UMass Memorial Hospital, Worcester, MA USA; 31 Department of Neuroradiology, Ospedale San Raffaele, Milan, Italy; 32 Department of Neurosurgery, University of Miami, Miami, FL USA; 33 Department of Neuroradiology, Valley Baptist Neuroscience Institute, Harlingen, TX USA; 34 Deparments of Neurosurgery and Radiology, University of Alabama at Birmingham, Birmingham, AL USA; 35 Department of Diagnostic and Interventional Neuroradiology, University Medical Center Hamburg-Eppendorf, Hamburg, Germany; 36 Department of Neuroradiology, University Hospital of Basel, Basel, Switzerland; 37 Departments of Radiology and Neurosurgery, Mayo Clinic, Jacksonville, FL USA; 38 Department of Neuroradiology, University of Texas Health Science Center at Houston, Houston, TX USA; 39 Department of Radiology, Division of Neuroimaging and Neurointervention, Stanford University School of Medicine, Stanford, CA USA

**Keywords:** Aneurysms, Brain, Follow-up, Incomplete occlusion, Woven EndoBridge

## Abstract

While the Woven EndoBridge (WEB) device has transformed the treatment of wide-neck intracranial aneurysms, incomplete occlusion remains a significant challenge requiring better understanding of contributing factors. A retrospective analysis was conducted on multicenter data from patients who underwent WEB device treatment for intracranial aneurysms between January 2011 and December 2022. Using machine learning models, Cox regression, and time-stratified analyses, we evaluated factors associated with persistent incomplete occlusion, defined as non-improving Raymond-Roy Occlusion Classification grade 2 or 3 at final follow-up. Among 813 patients (607 with < 24 months follow-up, 206 with ≥ 24 months), machine learning analysis identified aneurysm height, Acom location, neck diameter, and pretreatment mRS as predictors of persistent incomplete occlusion. On Cox regression. larger aneurysm neck diameter (HR 1.13, 95% CI 1.01–1.27, *p* = 0.027) and height (HR 1.14, 95% CI 1.02–1.26, *p* = 0.017), and radial access (HR 2.68, 95% CI 1.76–4.07, *p* < 0.001) increased, while posterior circulation location (HR 0.56, 95% CI 0.37–0.84, *p* = 0.005) decreased the risk of persistent incomplete occlusion. Time-stratified analysis revealed that in short-term follow-up (< 24 months), larger aneurysm neck diameter (OR 1.28, 95% CI 1.08–1.52, *p* = 0.004) increased the risk of incomplete occlusion. In long-term follow-up (≥ 24 months), smoking (OR 2.69, 95% CI 1.04–7.00, *p* = 0.04), higher pre-treatment mRS (OR 1.78, 95% CI 1.15–2.76, *p* = 0.009), and immediate flow stagnation (OR 0.33, 95% CI 0.11–0.96, *p* = 0.04) increased, while older age (OR 0.94, 95% CI 0.90–0.98, *p* = 0.002) and WEB-DL (OR 0.06, *p* < 0.001) and SLS devices (OR 0.02, *p* = 0.003) decreased the risk of persistent incomplete occlusion. Aneurysm characteristics and device type significantly influence long-term WEB treatment outcomes.

**Supplementary information:**

The online version contains supplementary material available at 10.1007/s10143-025-03439-8.

## Introduction

Intracranial wide-neck saccular aneurysms pose significant challenges in neurovascular treatment, traditionally requiring complex surgical or endovascular approaches with higher morbidity [20, 23]. The advent of endosaccular flow disruptors, particularly the Woven EndoBridge (WEB) device, has transformed their management [14]. The WEB device, designed specifically for wide-neck bifurcation aneurysms, provides a minimally invasive alternative to surgical clipping and stent-assisted coiling by effectively modifying intra-aneurysmal flow while preserving parent vessel patency [2, 4, 9, 15].

Despite its demonstrated efficacy, a significant challenge has emerged regarding incomplete occlusion rates. Studies have reported varying degrees of incomplete occlusion, with Cognard and Januel[7] observing remnants and recurrences in up to 71.5% of treated aneurysms in their series. Herbreteau et al.[18] found that device shape modification occurred in 31.6% of cases, potentially affecting occlusion rates. The WEBCAST and French Observatory studies reported complete occlusion rates of only 52.9%, with adequate occlusion achieved in 79.1% of cases at one year [16, 19].

This persistent incomplete occlusion phenomenon remains a significant challenge in ensuring durable aneurysm treatment and poses risks for recurrence and potential hemorrhage [17, 18, 21]. Multiple factors may contribute to incomplete occlusion, including device sizing, compression phenomena, and aneurysm characteristics, though their relative importance remains unclear [17, 18, 21].

This study aims to identify predictors of persistent incomplete occlusion following WEB deployment in a large multicenter cohort and seeks to optimize device use and improve long-term outcomes for challenging intracranial aneurysms.

## Materials and methods

### Patient dataset and variables

Our research retrospectively examines patient data from 36 hospitals across North and South America, Asia, Europe, and Australia, as part of the WorldWideWeb consortium tracking intracranial saccular aneurysms treated with the WEB device. Institutional Review Board (IRB) approval was obtained from each participating institution, which waived patient consent given the retrospective design, and the study included adults (≥ 18 years) treated between January 2011 and December 2022, regardless of aneurysm location or rupture status. Collected variables included demographics (age, gender, smoking status—analyzed both as an ordinal scale: never [0], former [1], current [2], and as binary: ever vs. never smoked), aneurysm characteristics (location, neck width, maximal diameter, height, width, daughter sac presence, dome-originating branch, bifurcation site, prior interventions, partial thrombosis), clinical presentation (symptoms, SAH history), treatment urgency (categorized as elective [≥ 2 weeks], subacute [24 h to < 2 weeks], and acute [within 24 h]), angiographic outcomes (initial and follow-up Raymond-Roy Occlusion Classification [RROC] grades assessed via DSA, CTA, or MRA; initial and final occlusion statuses; retreatments), procedural details (access, adjunctive devices, complications), and clinical outcomes (Hunt-Hess grade, pre-treatment modified Rankin Scale [mRS]).

The primary outcome of interest was residual incomplete aneurysm occlusion, defined as a non-improving RROC grade of 2 or 3 on the final imaging follow-up. Improved occlusion was defined as a change in RROC grade from 2 to 1 or from 3 to 2 or 1. Cases with stable grade 1 or worsening RROC scores were excluded to focus on predictors of persistent incomplete occlusion. Stable grade 1 indicates complete occlusion, offering no insight into factors for improvement. Worsening scores were considered beyond the scope of this study since they may suggest recurrence or other mechanisms.

### Statistical analysis

Univariate analyses were conducted using SciPy 1.6.2 in Python 3.9 to compare demographic and clinical features across patient cohorts. Continuous variables were summarized as medians with interquartile ranges (IQR), and categorical variables as frequencies and percentages. The Kruskal–Wallis test assessed non-normally distributed continuous variables, while the Chi-Square test was used for categorical variables. Statistical significance was set at *p* < 0.05.

#### Machine learning modeling and analysis

Machine learning models were developed as the primary analytical approach to predict persistent incomplete occlusion, with a dataset split into training (75%) and testing (25%) subsets, validated using tenfold cross-validation on the training subset. Feature processing included min–max scaling to normalize the feature set to a 0–1 range, optimizing the dataset for algorithmic learning. To improve model clarity and interpretability, dimensionality was reduced using the Maximum Relevance—Minimum Redundancy (MRMR) technique, filtering out the top 25% of significant features. Algorithms including Decision Tree, Gaussian Naïve Bayes, Multilayer Perceptron, K-Nearest Neighbors, Random Forest, Bagging Classifier, Gradient Boosting, and CatBoost were employed, with hyperparameter optimization via grid search. Model performance on the test set was evaluated using metrics like ROC-AUC, accuracy, F1 score, precision, and recall. The best model was further analyzed using Shapley Additive Explanations (SHAP) to clarify feature impacts, with detailed interpretations and case-specific force plots providing clinical insights into immediate occlusion status post-WEB deployment.

#### Secondary time-to-event analysis

As a secondary analysis to validate the machine learning findings, we conducted a Cox proportional hazards regression analysis. This multivariable analysis, performed in Stata Version 17.0, included covariates identified as important by the machine learning models. Results were reported as hazard ratios (HRs) with 95% Confidence Intervals (CIs). The proportional hazards assumption was tested using Schoenfeld residuals, showing no significant violations (global test *p* > 0.05). Kaplan–Meier curves were generated to visualize survival probability over time, showing no significant difference between ruptured and unruptured aneurysms (*p* > 0.05), obviating the need for further stratification by SAH status. A forest plot was generated to display HRs with 95% CIs for the covariates. Additionally, to examine time-dependent effects, follow-up periods were categorized as mid/short-term (< 24 months) or long-term (≥ 24 months), and a multivariable logistic regression was conducted to identify predictors of persistent incomplete occlusion at these timepoints, with odds ratios (OR) and 95% CIs reported.

## Results

### Patient cohort and univariable analysis

A total of 813 patients were included, with 607 having short/mid-term follow-up (< 24 months) and 206 with long-term follow-up (≥ 24 months). The median radiographic follow-up duration was similar between groups with persistent incomplete occlusion (14 months, IQR: 6–21) and improved occlusion (14 months, IQR: 7–24), with no significant difference (*p* = 0.08) (Table 1). Median age was slightly higher in the persistent incomplete occlusion group (61 vs. 59 years, *p* = 0.06), but not statistically significant. Gender distribution and smoking status also showed no significant differences (*p* = 0.12 and *p* = 0.56/0.40, respectively). Aneurysm location at bifurcation sites was similar between improved occlusion and persistent incomplete occlusion groups (80.4% vs 81.0%, *p* = 0.87), and between anterior and posterior circulations (80.7% vs 79.9%, *p* = 0.81). Ruptured aneurysm incidence was similar between groups (28.1% vs. 23.2%, *p* = 0.16), and pretreatment mRS scores were comparable (median score of 0, *p* = 0.19). Significant differences were observed in aneurysm characteristics. Persistent incomplete occlusion was associated with larger neck size (4.3 mm vs. 4.0 mm, *p* < 0.001), maximal diameter (7.0 mm vs. 6.5 mm, *p* < 0.001), and width (6.0 mm vs. 5.39 mm, *p* < 0.001). Anterior communicating artery complex (Acom) aneurysms were more frequent in the persistent incomplete occlusion group (33.8% vs. 25.2%, *p* = 0.02), while posterior circulation aneurysms were less frequent (14.3% vs. 20.6%, *p* = 0.046). Radial access trended higher in the persistent incomplete occlusion group (14.8% vs. 10.1%, *p* = 0.07). Retreatment was more common in the persistent incomplete occlusion group (21.4% vs. 4.8%, *p* < 0.001), while immediate flow stagnation did not differ significantly (*p* = 0.18).

**Table 1:** Baseline characteristics of patients and aneurysms treated with WEB device and technical characteristics of treatment stratified by occlusion status

Variable name	All 813	Improved occlusion 603 (74%)	Incomplete Occlusion 210 (16%)	*P* value
	*n* (%)	*n* (%)	*n* (%)	
Age (years), median (IQR)	60.0 (52.0, 68.0)	61.0 (52.0, 68.0)	59.0 (51.0, 67.0)	0.06
Gender (Male)	231 (28.4%)	162 (26.9%)	69 (32.9%)	0.12
Smoking	1 (0.0, 2.0)	1 (0.0, 2.0)	1.0 (0.0, 2.0)	0.56
Ordinal^#^, median (IQR)	256 (31.5%)	190 (31.5%)	66 (31.4%)	0.51
Current	202 (24.8%)	144 (23.9%)	58 (27.6%)	0.51
Former	355 (43.7%)	269 (44.6%)	86 (41.0%)	0.51
Never	458 (56.3%)	334 (55.4%)	124 (59.0%)	0.4
Ever smoked	183 (22.5%)	130 (21.6%)	53 (25.2%)	0.31
Ruptured Aneurysms	199 (24.5%)	140 (23.2%)	59 (28.1%)	0.16
Pretreatment mRS, median (IQR)	0 (0.0, 0.0)	0 (0.0, 0.0)	0.0 (0.0, 1.75)	0.19
Treatment Urgency Level^*^	60.0 (52.0, 68.0)	61.0 (52.0, 68.0)	59.0 (51.0, 67.0)	0.33
Elective	614 (75.5%)	463 (76.8%)	151 (71.9%)	
≥ 2 weeks	14 (1.7%)	8 (1.3%)	6 (2.9%)	
≤ 2 weeks	19 (2.3%)	13 (2.2%)	6 (2.9%)	
Acute	166 (20.4%)	119 (19.7%)	47 (22.4%)	
Location				
Posterior circulation	154.0 (18.9%)	124 (20.6%)	30 (14.3%)	0.046
AICA	1 (0.1%)	1 (0.2%)	0 (0.0%)	> .99
Anterior choroidal	3 (0.4%)	3 (0.5%)	0 (0.0%)	0.72
ICA	62 (7.6%)	44 (7.3%)	18 (8.6%)	0.65
Ophthalmic	11 (1.4%)	10 (1.7%)	1 (0.5%)	0.35
PICA	16 (2.0%)	12 (2.0%)	4 (1.9%)	> .99
Pcom	50 (6.2%)	38 (6.3%)	12 (5.7%)	0.88
SCA	5 (0.6%)	5 (0.8%)	0 (0.0%)	0.42
VA	6 (0.7%)	5 (0.8%)	1 (0.5%)	0.96
Aneurysm characteristics				
Aneurysm count (including nontreated)				
Bifurcation aneurysm	655 (80.6%)	485 (80.4%)	170 (81.0%)	0.94
Aneurysm maximum diameter (mm), median (IQR)	6.7 (5.4, 8.3)	6.5 (5.27, 8.0)	7.0 (6.0, 9.0)	< .001
< 10 mm	687 (84.5%)	526 (87.2%)	161 (76.7%)	< .001
≥ 10 mm	126 (15.5%)	77 (12.8%)	49 (23.3%)	< .001
Aneurysm height (mm), median (IQR)	5.7 (4.56, 7.3)	5.6 (4.415, 7.0)	6.2 (5.0, 8.775)	< .001
Aneurysm width (mm), median (IQR)	5.6 (4.49, 7.0)	5.39 (4.3, 7.0)	6.0 (5.0, 7.675)	< .001
Aneurysm neck (mm), median (IQR)	4.0 (3.2, 5.02)	4.0 (3.1, 5.0)	4.3 (3.3925, 5.875)	< .001
Daughter sac	213 (26.2%)	155 (25.7%)	58 (27.6%)	0.65
Branch from aneurysm	117 (14.4%)	83 (13.8%)	34 (16.2%)	0.45
Partially thrombosed aneurysm	13 (1.6%)	7 (1.2%)	6 (2.9%)	0.17
Prior treatment	60 (7.4%)	43 (7.1%)	17 (8.1%)	0.75
WEB type				0.02
SL	686 (84.4%)	516 (85.6%)	170 (81.0%)	0.13
SLS	100 (12.3%)	73 (12.1%)	27 (12.9%)	0.87
DL	27 (3.3%)	14 (2.3%)	13 (6.2%)	0.01
Radial access	92 (11.3%)	61 (10.1%)	31 (14.8%)	0.07
Use of adjunct device	50 (6.2%)	35 (5.8%)	15 (7.1%)	0.59
Thromboembolic complications	55 (6.8%)	39 (6.5%)	16 (7.6%)	0.67
Radiographic follow-up, median (IQR)	14 (14, 24)	14 (7, 24)	14 (6, 21)	0.08
Short/Mid-term Follow-up (< 24 months)	607 (74.7%)	444 (73.6%)	163 (77.6%)	0.25
Long-term Follow-up (≥ 24 months)	206 (25.3%)	159 (26.4%)	47 (22.4%)	
Retreatment	74 (9.1%)	29 (4.8%)	45 (214%)	< .001

^**#**^Smoking was treated as an ordinal variable in the order 0 “never smoker” | 1 “former smoker” | 2 “current smoker”

^*^Treatment urgency level was categorized in relation to treatment time in 4 groups as follows; elective, chronic (< 2 weeks), subacute (≥ 2 weeks – 24 h), and acute (within 24 h). This variable was treated as a continuous variable respectively in the order 0 | 1 | 2 | 3

*AICA;* Anterior inferior cerebellar artery, *DL;* Dual layer, *ICA;* Internal carotid artery, *IQR;* Interquartile range, *mRS;* modified Rankin Scale, *Pcom;* posterior communicating artery, *PICA;* Posterior inferior cerebellar artery, *SCA;* Superior cerebellar artery, *SL;* Single laye, *SLS;* Single layer spherical, *VA;* Vertebral artery, *WEB;* Woven EndoBridge

### Predictors of persistent incomplete occlusion

#### Machine learning model performance and feature importance

**Fig. 1:**
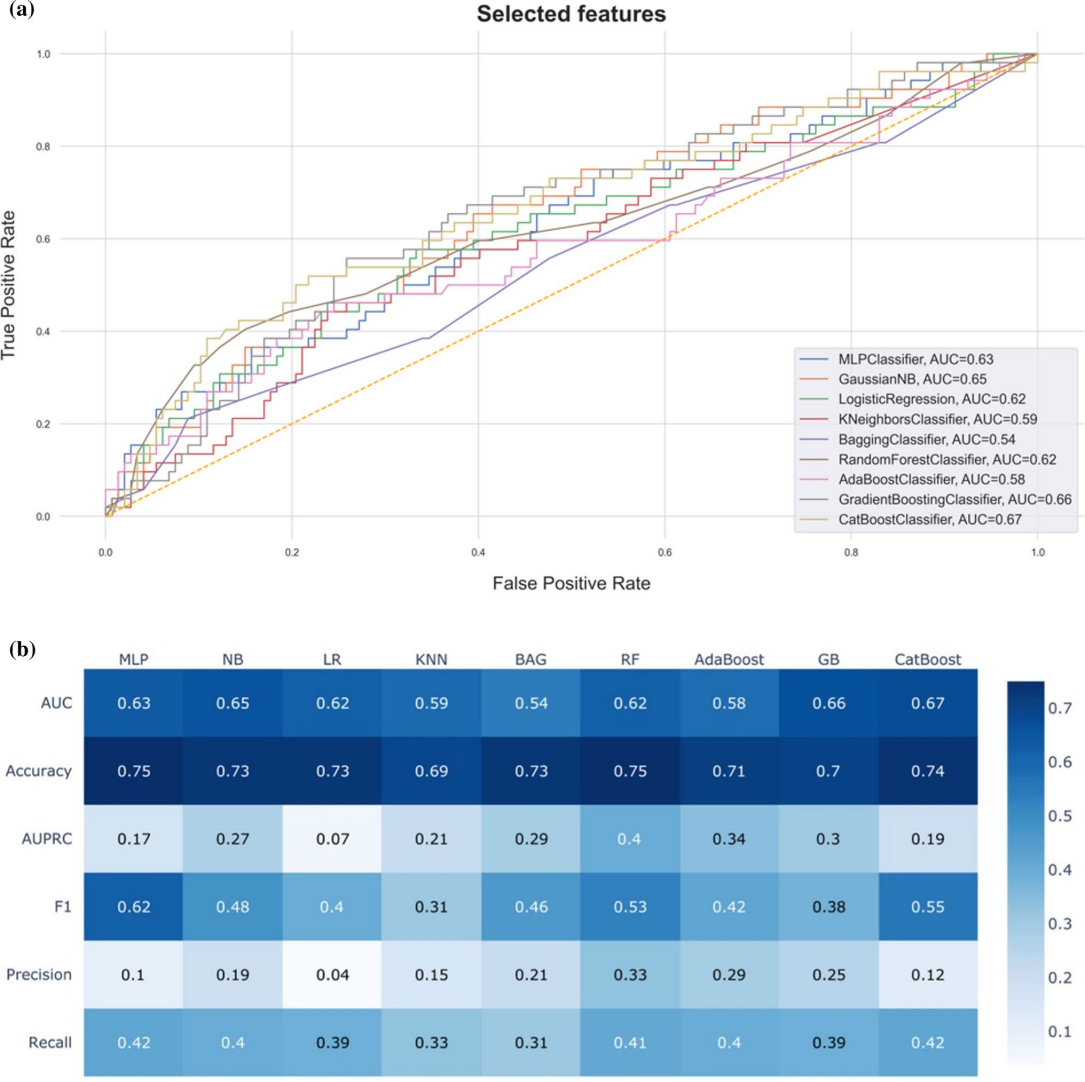
**A** Receiver operating characteristic curves (**B**) and evaluation metrics matrix for predicting persistent incomplete occlusion following WEB treatment of cerebral aneurysm

**Fig. 2:**
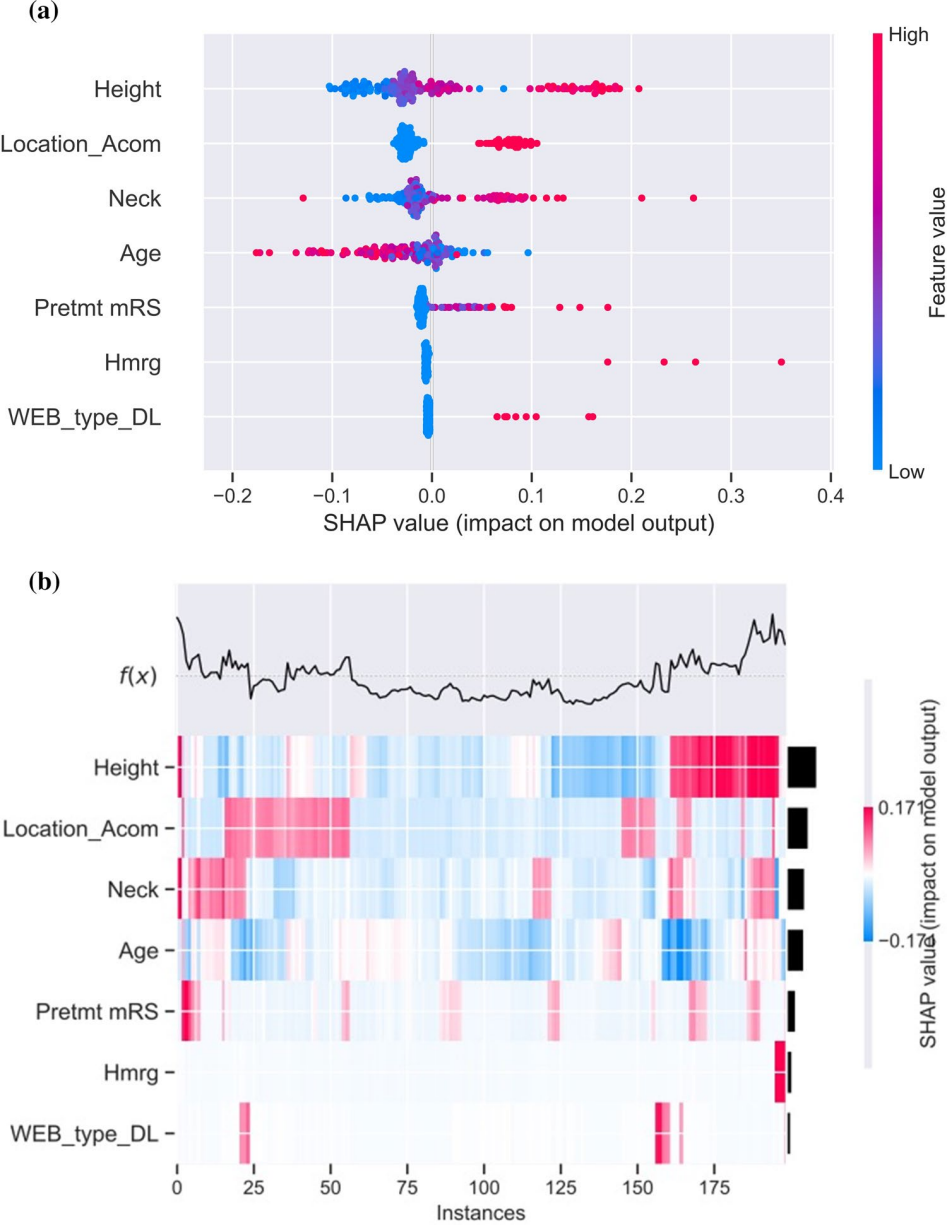
Interpretation of the best performing predictive model applied to the test set instances. **A** SHAP summary plot with the colors signifying feature value and (**B**) SHAP heatmaps of the 10 most influential features, where the red and blue refer to positive and negative SHAP values

**Fig. 3:**
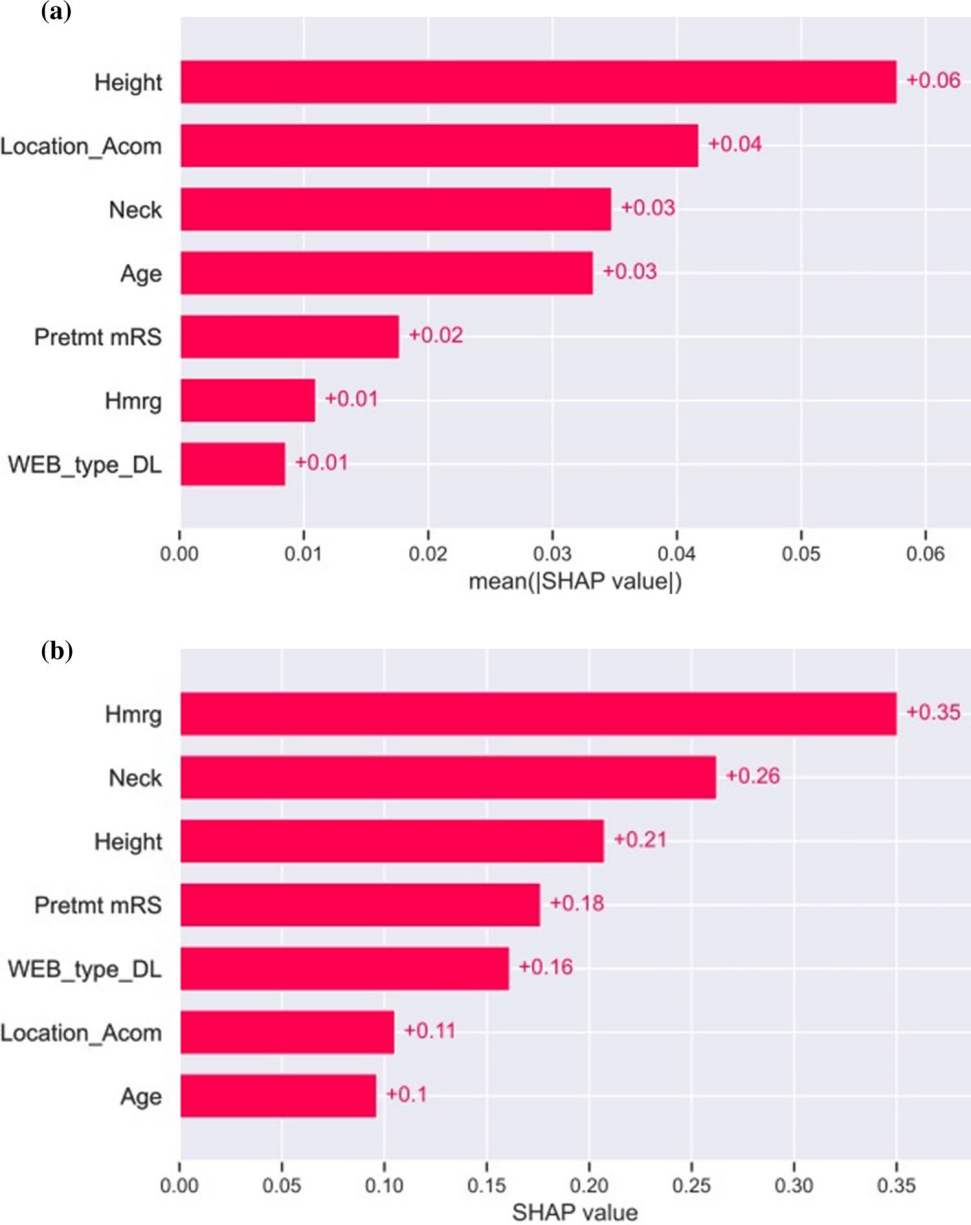
SHAP bar plot of feature importance with (**A**) the mean absolute SHAP value of each feature and (**B**) the maximal absolute SHAP value of each feature of the test set best-performing model

The CatBoost classifier emerged as the superior model for predicting persistent incomplete occlusion. The model achieved an AUROC of 0.67, accuracy rate of 74%, area under the precision-recall curve of 0.19, F1-score of 55, precision (1-specificity) of 0.12, and recall (sensitivity) of 0.42. SHAP analysis identified key predictive features, ranked by importance: aneurysm height, Acom location, neck diameter, age, pretreatment mRS, hemorrhagic complication, and DL WEB type. All features exhibited positive correlation with persistent incomplete occlusion, except for age, which demonstrated negative correlation. Model performance comparisons and detailed metrics are presented in Fig. 1A and B, with feature importance visualizations in Figs. 2 and 3.

#### Secondary time-to-event analysis

**Fig. 4:**
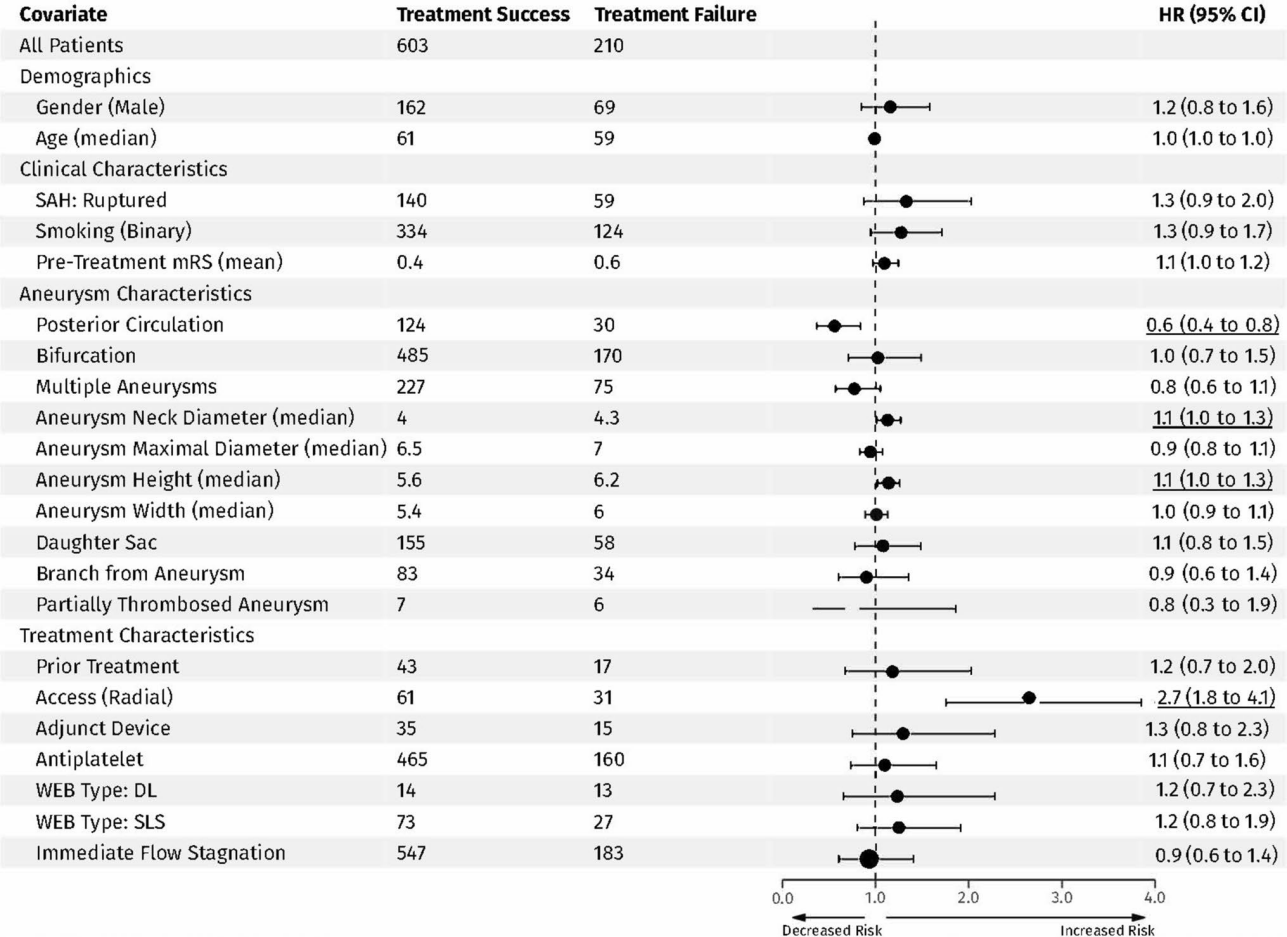
Cox regression analysis forest plot illustrates the predictors of persistent incomplete occlusion over time. *Significant associations were underlined

Cox regression analysis demonstrated several significant predictors of persistent incomplete occlusion (Fig. 4). Posterior circulation aneurysms were associated with lower risk (HR 0.56, 95% CI 0.37–0.84,* p* = 0.005), while larger aneurysm neck diameter (HR 1.13, 95% CI 1.01–1.27, *p* = 0.027), aneurysm height (HR 1.14, 95% CI 1.02–1.26, *p* = 0.017), and radial access (HR 2.68, 95% CI 1.76–4.07, *p* < 0.001) increased risk. WEB devices of type DL (HR 1.23, 95% CI 0.66–2.28, *p* = 0.52) and immediate flow stagnation (HR 0.93, 95% CI 0.61–1.41, *p* = 0.74) showed no significant effect. Other factors, including patient demographics, smoking status, and most aneurysm characteristics, did not significantly influence risk.

#### Time-dependent effects

Analysis stratified by follow-up duration revealed distinct predictors (Table 2). In long-term follow-up, age demonstrated a protective effect (OR 0.94, 95% CI 0.90–0.98, *p* = 0.002), while smoking (OR 2.69, 95% CI 1.04–7.00, *p* = 0.04) and higher pre-treatment mRS (OR 1.78, 95% CI 1.15–2.76, *p* = 0.009) increased odds of persistent incomplete occlusion. Posterior circulation aneurysms showed reduced odds in both follow-up periods (short/mid-term OR 0.56, 95% CI 0.33–0.95, *p* = 0.03; long-term OR 0.20, 95% CI 0.05–0.74, *p* = 0.02). Larger aneurysm neck diameter increased odds in short/mid-term (OR 1.28, 95% CI 1.08–1.52, *p* = 0.004), while radial access significantly increased odds in long-term follow-up (OR 17.29, 95% CI 2.56–116.78, *p* = 0.003). WEB devices DL and SLS demonstrated reduced odds in long-term follow-up compared to SL (DL: OR 0.06, 95% CI 0.01–0.28, *p* < 0.001; SLS: OR 0.02, 95% CI 0.00–0.26, *p* = 0.003), as did immediate flow stagnation (OR 0.33, 95% CI 0.11–0.96, *p* = 0.04).

**Table 2:** Multivariable binomial logistic regression model to evaluate the predictors of persistent incomplete occlusion within follow-up cohorts

Covariate	Short/Mid-term follow-up (< 24 months)	*P* value	Long-term follow-up (≥ 24 months)	*P* value
	OR (95% CI)		OR (95% CI)	
Demographics				
Gender (Male)	1.24 (0.81, 1.88)	0.32	0.85 (0.33, 2.20)	0.74
Age (median)	0.99 (0.97, 1.01)	0.22	0.94 (0.90, 0.98)	0.002
Clinical characteristics				
SAH: Ruptured	1.53 (0.91, 2.57)	0.11	0.33 (0.07, 1.49)	0.15
Smoking (Binary)	1.02 (0.69, 1.50)	0.94	2.69 (1.04, 7.00)	0.04
Pre-Treatment mRS (mean)	1.08 (0.91, 1.29)	0.38	1.78 (1.15, 2.76)	0.009
Aneurysm characteristics				
Posterior circulation	0.56 (0.33, 0.95)	0.03	0.20 (0.05, 0.74)	0.02
Bifurcation	1.22 (0.75, 1.99)	0.42	0.59 (0.20, 1.80)	0.36
Multiple aneurysms	0.81 (0.53, 1.22)	0.31	0.78 (0.32, 1.92)	0.59
Aneurysm neck diameter (median)	1.28 (1.08, 1.52)	0.004	1.27 (0.87, 1.84)	0.21
Aneurysm maximal diameter (median)	1.00 (0.83, 1.20)	0.99	1.26 (0.78, 2.04)	0.34
Aneurysm height (median)	1.17 (1.01, 1.36)	0.04	1.11 (0.80, 1.55)	0.52
Aneurysm width (median)	0.93 (0.79, 1.09)	0.36	1.00 (0.66, 1.51)	0.98
Daughter Sac	0.92 (0.59, 1.42)	0.70	0.97 (0.33, 2.81)	0.95
Branch from aneurysm	1.44 (0.86, 2.44)	0.17	0.56 (0.13, 2.49)	0.45
Partially thrombosed aneurysm	1.12 (0.27, 4.71)	0.87	0.58 (0.02, 14.30)	0.74
Treatment characteristics				
Prior treatment	0.80 (0.36, 1.79)	0.59	4.30 (0.96, 19.28)	0.06
Radial access	1.41 (0.82, 2.42)	0.22	17.29 (2.56, 116.78)	0.003
Adjunct device	0.82 (0.38, 1.76)	0.61	2.03 (0.22, 18.98)	0.54
Antiplatelet	1.46 (0.87, 2.46)	0.16	0.63 (0.19, 2.09)	0.45
WEB type: DL	0.92 (0.25, 3.30)	0.90	0.06 (0.01, 0.28)	< 0.001
WEB type: SLS	1.24 (0.31, 4.94)	0.76	0.02 (0.00, 0.26)	0.003
Immediate flow stagnation	0.70 (0.36, 1.36)	0.29	0.33 (0.11, 0.96)	0.04

*CI;* Confidence interval, *DL;* Dual layer, *mRS;* modified Rankin Scale, *OR;* Odds ratio, *SAH;* Subarachnoid hemorrhage, *SLS;* Single layer spheric, *WEB;* Woven EndoBridge

## Discussion

This large multinational cohort study comprehensively analyzes factors influencing the long-term radiographic success of intracranial saccular aneurysms treated with the WEB device. The results indicate that certain factors, such as larger aneurysm neck diameters and radial access, were associated with a higher risk of incomplete occlusion. In contrast, aneurysms in the posterior circulation and those achieving immediate post-procedural flow stagnation showed a lower risk of persistent incomplete occlusion. When adjusted for angiographic follow-up, WEB SLS and DL types were deemed protective against persistent incomplete occlusion compared to WEB SL in the long-term follow-up cohort. These findings underscore the critical role of precise device selection, procedural planning, and continuous monitoring to optimize patient outcomes. Machine learning’s ability to handle high-dimensional data enabled analysis of specific locations like Acom, while traditional statistical approaches helped validate broader anatomical distinctions between anterior and posterior circulation outcomes. The favorable results in posterior circulation aneurysms likely reflect their better dome-to-neck ratios, and less complex perforator patterns compared to anterior circulation aneurysms. The challenges with radial access likely stem from technical factors including reduced catheter stability and more complex navigation of tortuous anatomy, particularly during operators’ learning curves.

The results are consistent with previous studies highlighting the impact of the aneurysm morphology and device-related factors on occlusion outcomes. For instance, Kewlani et al. demonstrated that larger aneurysm neck size and suboptimal device selection are associated with a higher likelihood of incomplete occlusion [12]. Additionally, Fortunel et al. reported that larger aneurysm sizes and more complex morphologies increase the likelihood of incomplete occlusion, underscoring the need for careful device sizing and potential adjunctive techniques in such cases [10]. Our findings also align with Alpay et al., who noted that aneurysm morphology, particularly wide-neck aneurysms, poses challenges in achieving stable thrombosis, impacting occlusion success [1]. Furthermore, Pierot et al. found that posterior circulation aneurysms had better long-term occlusion rates than anterior circulation aneurysms (such as Acom and ICA terminus), which mirrors our results [18].

Our angiographic follow-up-adjusted survival and logistic regression analyses further revealed the changing nature of factors influencing occlusion stability over time. Notably, older age emerged as a protective factor for long-term outcomes, which may reflect age-related differences in aneurysm biology and hemodynamics that promote stable thrombosis [8]. With aging, changes in vascular wall composition, reduced flow dynamics, and an increased propensity for progressive thrombosis could contribute to more durable occlusion [8]. Conversely, higher pre-treatment mRS scores were associated with poorer long-term results, likely reflecting a combination of underlying vascular health conditions and pre-existing neurological deficits that not only impair initial recovery but also hinder long-term vessel remodeling and healing [11]. Interestingly, although the WEB-DL device was initially linked to worse angiographic outcomes, it showed—along with WEB SLS—a significantly lower risk of incomplete occlusion over the long term. This may be due to the dual-layer structure of the WEB-DL, which likely stabilizes more effectively within the aneurysm, supporting delayed but sustained thrombosis and endothelialization [13, 17]. On the other hand, WEB SLS appears to offer better durability than WEB SL, which is more prone to compaction, given its design [3]. Interestingly, radial access was associated with incomplete occlusion in our cohort, likely due to factors such as limited catheter stability, difficulties in navigating tortuous anatomy, and a steep operator learning curve which might have affected the optimal deployment of the WEB device and, consequently, long-term angiographic outcomes [5, 6, 22].

The temporal evolution of device performance, particularly regarding WEB-DL and SLS, adds important nuance to our understanding of device selection. The emergence of smoking as a risk factor specifically in long-term follow-up suggests its effects may manifest through delayed mechanisms such as impaired endothelialization or increased inflammatory responses. The need for continuous monitoring and follow-up is emphasized by Pierot et al., who noted that while the long-term efficacy of the WEB device is generally good, there is still a risk of device compression and aneurysm recurrence over time [21]. This calls for regular follow-up, particularly in patients with more complex aneurysms, to detect and manage any recurrence early. Similarly, Pierot et al. reported that most neck remnants observed in the mid-term remained stable or improved at long-term follow-up, suggesting that while aggressive retreatment is not always necessary due to the potential for progressive improvement in occlusion status, consistent imaging and clinical evaluations are crucial to prevent adverse outcomes [17]. The true dilemma is that it is still unclear if radiographic failure translates to a clinically meaningful risk for bleed, which is the primary goal in aneurysm treatment. There are very few reports discussing the bleed risk of aneurysms treated with WEB with incomplete occlusion on angiographic follow up.

Furthermore, the application of machine learning models, such as the CatBoost classifier, added value by improving occlusion outcomes’ predictive accuracy and interpretability. Although the model demonstrated moderate performance (AUROC of 0.67 and accuracy of 74%), it effectively identified key predictors, including aneurysm height, Acom location, neck diameter, and WEB-DL type. Using SHAP values provided deeper insights into the interplay between patient characteristics and procedural factors, enhancing clinical decision-making.

Overall, this study identifies key factors influencing outcomes in intracranial aneurysm treatment with devices like the WEB-DL. Differences in risk factors for persistent incomplete occlusion between short/mid-term and long-term follow-ups suggest a need for customized follow-up plans. Important predictors include patient age, smoking, aneurysm characteristics (neck size, height), radial access, and device type. These findings support the need for long-term monitoring to assess occlusion status and refine treatment strategies.

## Limitations and strengths

This study has some limitations, mainly due to its retrospective design, which can lead to selection and information bias and may not account for all influencing factors. The wide variety of patients from several hospitals with different treatment protocols and healthcare settings may improve overall generalizability, but it remains challenging to apply these findings to individual centers. Differences in how data were collected and missing data could also affect the accuracy of our models. A key limitation is our inability to account for pre-existing anticoagulation status, though we did analyze post-procedural antiplatelet therapy (77.1% vs 76.2%, *p* = 0.78). This is important because pre-existing blood thinning medications could potentially influence both initial thrombosis and subsequent aneurysm occlusion rates, affecting our assessment of treatment outcomes. The use of different imaging modalities (DSA, CTA, MRA) for follow-up assessment could introduce measurement variability, though our results remained consistent across modalities. Additionally, without external validation, our machine-learning models may not be fully reliable in other settings. The device-to-aneurysm volume (DAV) ratio, recognized as a significant predictor of occlusion success, could not be assessed in our study [12]. Despite these limitations, the study has several strengths. The large and varied dataset helps provide a broader view of treatment outcomes. Including many types of aneurysms and treatment approaches may provide a more thorough picture of what influences success. Advanced machine learning techniques, like feature selection and SHAP analysis, allow for better identification of important predictors. Moreover, the long follow-up period offers valuable insights into the durability of the WEB device, aiding in clinical decisions.

## Conclusions

The WEB device is a significant advancement for treating wide-neck bifurcation aneurysms but achieving durable occlusion for all patients remains a key challenge. Our findings emphasize the need for precise patient selection, accurate device sizing, and consistent follow-up monitoring. Factors such as aneurysm neck size, access route, location, and device type (DL vs. SLS) are critical for successful outcomes. The efficacy of the WEB device depends on a tailored approach that considers specific patient and aneurysm characteristics. Ongoing research and technological improvements are necessary to enhance the device’s long-term effectiveness and optimize patient outcomes in intracranial aneurysm treatment.

## Supplementary information

Below is the link to the electronic supplementary material.

ESM 1 (DOCX 32.9 KB)

## Data Availability

Data is available upon the request and approval of the corresponding  authors and institutions.

## Declarations

### Clinical trial

Clinical trial number: not applicable.
